# Addressing the consequences of the corporatization of reproductive medicine

**DOI:** 10.1093/medlaw/fwae018

**Published:** 2024-07-25

**Authors:** Sara A Attinger, Emily Jackson, Isabel Karpin, Ian Kerridge, Ainsley J Newson, Cameron Stewart, Lucy van de Wiel, Wendy Lipworth

**Affiliations:** Faculty of Medicine and Health, Sydney School of Public Health, Sydney Health Ethics, The University of Sydney, Sydney, Australia; Department of Philosophy, Macquarie University, Macquarie Park, Australia; Law School, London School of Economics and Political Science, UK; Faculty of Law, University of Technology Sydney, Australia; Faculty of Medicine and Health, Sydney School of Public Health, Sydney Health Ethics, The University of Sydney, Sydney, Australia; Department of Philosophy, Macquarie University, Macquarie Park, Australia; Haematology Department, Royal North Shore Hospital, St Leonards, Australia; Faculty of Medicine and Health, Sydney School of Public Health, Sydney Health Ethics, The University of Sydney, Sydney, Australia; Sydney Health Law, Sydney Law School, University of Sydney, Sydney, Australia; Department of Global Health & Social Medicine, Kings College London, UK; Faculty of Medicine and Health, Sydney School of Public Health, Sydney Health Ethics, The University of Sydney, Sydney, Australia; Department of Philosophy, Macquarie University, Macquarie Park, Australia

**Keywords:** assisted reproduction, assisted reproductive technologies, commercialization, corporatization, fertility treatment, reproductive choice

## Abstract

In Australia and the UK, commercialization and corporatization of assisted reproductive technologies have created a marketplace of clinics, products, and services. While this has arguably increased choice for patients, ‘choice’, shaped by commercial imperatives may not mean better-quality care. At present, regulation of clinics (including clinic–corporations) and clinicians focuses on the doctor–patient dyad and the clinic–consumer dyad. Scant attention has been paid to the conflicts between the clinic–corporation’s duty to its shareholders and investors, the medical profession’s duty to the corporations within which they practice, and the obligations of both clinicians and corporations to patients and to health systems. Frameworks of regulation based in corporate governance and business ethics, such as stakeholder models and ‘corporate social responsibility’, have well-recognized limits and may not translate well into healthcare settings. This means that existing governance frameworks may not meet the needs of patients or health systems. We argue for the development of novel regulatory approaches that more explicitly characterize the obligations that both corporations and clinicians in corporate environments have to patients and to society, and that promote fulfilment of these obligations. We consider mechanisms for application in the multi-jurisdictional setting of Australia, and the single jurisdictional settings of the UK.

## I. INTRODUCTION

Fertility has long been described as a ‘business’,^1^ and in recent decades the global marketplace for assisted reproductive technologies (ART) has grown, shifted and been shaped by processes of commercialization. This has led to profound changes in the web of relationships that shape the delivery of reproductive care, with commercialized clinic–corporations and investors, including private equity, gaining increasing power and influence. Given the impact of commercialization, it is important to critically examine the commercial and, more specifically, corporate forms that operate in this context, to assess their impact on quality of care and to determine *what* to regulate, *who* to regulate, and *how* to do so.

While we focus here on ART and reproductive choice, corporatization of clinical practice is not unique to ART^2^ and our analysis of the impacts of commercialization and corporatization will have broader relevance in other areas of health care. The development of ART in the UK and Australia does, however, encapsulate many relevant trends in corporatization and financialization, and forms of privatization and commercialization. Furthermore, fertility is a contested space in which there are ongoing debates about appropriate funding models, including in countries (such as the UK and Australia) where ART is supported by a mix of ‘public’ and ‘private’ funding and provision. ART is also subject to a higher level of regulation than many areas of medicine. ART is therefore a rich case study for understanding the impacts of commerce on patient care and the regulatory development this may necessitate.

In this article, we first describe the forms of commercial and corporate entities in assisted reproduction in Australia and the UK (Section II). In Section III, we consider the connections between commercialization, corporatization, and patient choice, and conclude that while processes of corporatization may increase patient choice, they may not do so in entirely unproblematic ways. In Section IV, we analyse some of the corporate models and strategies deployed in ART. We problematize reliance upon the doctor–patient dyad in regulation and show how there is a tension between the need to simultaneously separate the clinic and commercial entity, and enable them to influence each other. This tension between insulation and influence is at the heart of the regulatory problem. In Section V, we consider alternative mechanisms for governing corporations such as stakeholder corporate governance models, ‘corporate social responsibility’ and clinical governance, and their limitations in the ART context. In Section VI, we offer some potential ways forward, including mechanisms to bolster corporate accountability in the medical context and medical accountability in the corporate context.

## II. COMMERCIALIZATION AND CORPORATIZATION OF ART IN THE UK AND AUSTRALIA

### A. Terminology

For the purposes of this article, we use the term ‘corporatization’ to refer to the use of corporate forms to deliver health care, as distinct from other forms of private health care delivery, such as sole practice and partnerships. ‘Commercialization’ is a broader term we use to describe the process of providing health care for profit, including considerations of the products and services available, advertising, pricing, and competition. We also draw distinctions between various systems of financing and delivery of ART. From the financing perspective, public payer systems are those that either provide direct funding for ART services or pay via government-run insurance schemes.^3^ These can be contrasted with private payer systems where a patient pays directly or through their private health insurance. Some ‘hybrid’ systems include a combination of both public and private payer models, with governments partially subsidizing services and patients paying an additional amount. In terms of ART delivery, we can contrast a public-integrated model where government is both the funder and provider of ART services, public-contract models, where public payers contract with private health-care providers of ART to reimburse them for services, and private payer and provider ART, where the process of delivery does not involve government funds at all.^4^

Table 1 outlines a taxonomy of corporate and commercial entities in ART and Table 2 contains a taxonomy of terms concerning types of fertility clinics, based on ownership, service model and payment.

**Table 1:** Taxonomy of corporate and commercial entities in the fertility industry

**Category/Subcategory**	**Description/Example**
***(1) ART medical service providers***	*Corporate or commercial entities that provide medical services related to ART to patients.*
Fertility specialist medical practitioner	A medical practitioner that provides clinical fertility services, which may include fertility patient consultation, management of a cycle of treatment conducted in a fertility clinic, and relevant surgical interventions.
	E.g. A fertility specialist provides ART services at a private fertility clinic under a contract for services.
Fertility clinics	An organisation that typically offers (or otherwise facilitates) the following services in relation to fertility care: embryology and laboratory, diagnostics and imaging, pathology, day hospitals, egg or sperm freezing and storage and counselling.
	Some services may be provided by the clinic-company group or may be contracted out to other companies, depending on location and organisational structure.
	Some services may be provided by a separate corporate entity that is owned partly or wholly by a parent company.
	E.g. Monash IVF fertility clinics in Australia are part of Monash IVF Limited - a public holding company with 21 fertility clinics, 17 ultrasound clinics, three ARS service centres, 2 specialised diagnostics laboratories and 1 day hospital.^i^ (See also Table 2 *Taxonomy of fertility clinics*)
***(2) Support or ancillary service providers***	*Corporate or commercial entities that provide services that are integral to the delivery of ART treatment. May be suppliers to or within a fertility clinic or interact directly with individuals.*
Counsellors/psychologists	May be associated with a clinic or be independent. May be specialised eg genetic counsellors. Consultation with a counsellor may be a legal requirement for certain types of fertility treatment.
Donor and surrogacy agencies	Agencies that connect couples or individuals who need assisted reproductive services with prospective donors or surrogates. They may offer administrative and legal assistance to support such arrangements.
Sperm banks	Collect, store and distribute sperm.
Egg freezing services	Offer egg freezing and storage services. May also coordinate egg retrieval with other providers.
Diagnostics and imaging	Offer diagnostic tests and imaging services, including ultrasound and pathology.
Private day hospitals	Outpatient hospital facilities that may be used by medical professionals (for a fee) or provide services with contracted medical professionals.
Genetic testing laboratories	Offer services to test and screen individuals and embryos for indicators of genetic disorders.
***(3) Support or ancillary suppliers***	*Corporate or commercial entities that provide goods or services that are integral to the delivery of ART treatment.*
Fertility medication manufacturers	Produce drugs that are used to treat infertility.
Biotechnology and device manufacturers	Develop and/or sell technologies or devices for use in ART clinics.
Information technology providers	Develop and/or sell information technology products or services for use in ART clinics.
	eg management software or apps
***(4) Other health providers***	*Corporate or commercial entities that provide healthcare services that are not the clinical components of ART. May be suppliers to or within a fertility clinic or interact directly with individuals.*
Allied health providers	eg dieticians
Complementary and alternative medicine	eg ‘fertility naturopaths’
***(5) Professional financial management or investment***	*Corporate or commercial entities or organisations in the business of financial management and capital investment.*
Private equity firms	A financial or investment fund that invests in or acquires private companies. These organisations typically seek to takeover businesses with the goal of reorganising or growing that business and reselling the organisation at a profit. Private equity firms may also offer or facilitate venture capital financing, typically to a new or expanding enterprise.
***(6) Third party facilitators or brokers***	*Non-clinical third parties that provide goods or services that facilitate accessing ART treatment and related services*
Personal loan providers or brokers	Provide or arrange loan credit in exchange for fees and/or interest on amount borrowed.
Payment scheme providers	Offer financial products or consumer credit schemes that are accepted by clinics directly as payment methods.
	eg buy-now-pay-later schemes such as ‘Afterpay’ or ‘Zip’
Special financing agencies	Agencies that specialise in drafting and filing applications for accessing funding schemes for a fee.
	eg superannuation access brokers (Australia)
Health insurance companies	Companies that offer insurance plans that cover fertility treatment, including specialist fertility insurers.
‘Concierge’ agencies	Agencies that specialise in arranging access to or coordinating ART treatment and related services, which may include providing administrative support and legal assistance.
	eg online only or ‘virtual’ fertility ‘clinics’
Cross-border facilitators	Agencies that specialise in arranging access to or coordinating ART treatment and related services across borders, which may include providing administrative support and legal assistance. Cross-border care agencies typically offer services to individuals in jurisdictions where local services are not accessible for financial and/or legal reasons.^ii^
	eg donor and surrogacy agencies (see above), medical tourism agencies

i IBIS World, OD5091 Fertility Clinics in Australia Industry Report, **(**December 2020**)**.

ii It should be noted that these facilitators often operate at the boundaries of legality.

**Table 2:** Taxonomy of fertility clinics^i^

Clinic type	Sub-type/s: structure, ownership and financing	Service models	Payment
**(1) Public**	(1a) Owned and operated by public hospital or health service	Bulk-billing – no ‘gap’ cost to patient for government subsidised services, but some services not covered (eg day hospital fees) Free – no cost to patient for complete IVF cycle Mixed – treats public patients (i or ii) and private patients (see below)	Provides only treatment that is partially or wholly government subsidised.
**(2) Hybrid**	(2a) Offered as a public health service but provided by a private clinic ^ii^		Typically costs recovery only. Any surplus is reinvested into the clinic or public health system^iii^
	(2b) Joint venture between a private clinic and a public health organisation		
**(3) Commercial – Corporate chain – Publicly-traded**	(3a) Listed on the stock exchange (larger shareholders may include private investment firms and other companies)	‘Full service’ ‘Basic’, ‘low cost’ or ‘low profit’ Satellite or virtual clinic Boutique or specialty^iv^	Provides treatment that is partially government subsidised or patient self-funded.
**(4) Commercial – Corporate chain – Privately-held**	(4a) Owned wholly by private equity firm		Any surplus (profit) generated may be distributed to shareholders as dividends.
	(4b) Majority-owned by private investment firm or other company/ies		
	(4c) Majority-owned by doctors		
**(5) Commercial – Standalone**	(5a) Majority-owned by doctors		
	(5b) Institutionally-owned, e.g. by a university		
**(6) Private – Not for profit**	(6a) Constituted as trust, controlled by trustees	‘Full service’ ‘Basic’ or ‘low cost’ Satellite clinic Boutique or specialty^iv^	Provides treatment that is partially government subsidised or patient self-funded.
	(6b) Incorporated and limited by guarantee, controlled by members May or may not be classified as a charity.		Costs recovery only. Any surplus generated must be reinvested within the organisation.

i The legal distinction between shareholding and ownership is not relevant to this taxonomy and we use corporate shareholding as indicative of ownership for descriptive purposes only.

ii A public health service may ‘contract out’ the performance of some or all of the operations of the clinic to private clinic operators but retain control over pricing.

iii The corporate structure of the clinic indicates some aspects of how an organisation may be run, such as whether ‘surplus’ may be transferred out of the corporation to owners/shareholders as dividends. However, this does not always provide a complete picture of the company’s operations and rationale. For example, Westmead Fertility in Australia is owned by university, associated with a public health organisation and may be regarded as a ‘public’ clinic. It is, however, set up as a private company with shareholders (rather than, for example, a corporation limited by guarantee) and appears to generate some 'profit' from users (ie charges more than the cost of providing services to that patient) and to use those surplus funds within the clinic (i.e. a costs recovery model).

iv A clinic may focus on a particular type of fertility journey, such as same sex couples or donor and surrogacy services

### B. ART in the UK and Australia

In 1978, Louise Brown, the first IVF baby, was born in the UK. As ART began to be made available to the UK population through the NHS, private clinics also emerged, but they were viewed as an exception to the public-integrated model of ART. For example, the Warnock Report, whose recommendations formed the basis of the UK’s regulatory regime, acknowledged the existence of private clinics,^5^ but did not discuss the implications of a flourishing commercial market in private fertility treatment. Nevertheless, the following decades saw an enormous growth in private providers, with corresponding increases in commercialization and corporatization.

Currently the UK (population 67.3 million) has 107 clinics licensed by the Human Fertilization and Embryology Authority (HFEA) to provide fertility treatment, 62 per cent of which are privately owned rather than part of the National Health Service (NHS).^6^ It is routine for NHS clinics to treat a mixture of NHS and private patients, and, although there is substantial variation in NHS funding among the devolved nations (in 2021, 24 per cent of IVF cycles were NHS-funded in England, compared to 30 per cent in Wales and 58 per cent in Scotland),^7^ on average around 75 per cent of all cycles are self-funded by patients.^8^ Even among women under the age of 40 years, who are in theory eligible for NHS funding throughout the UK, 67 per cent of IVF cycles are privately funded. While the National Institute for Health and Care Excellence (NICE) recommends that the NHS should fund three full cycles of IVF for women under the age of 40, and one full cycle for women aged 40–42 years (who have not had IVF before and who do not have low ovarian reserve), in England, the majority of Integrated Care Boards provide one subsidized cycle only, to women under the age of 40 years.^9^

In contrast to the UK, Australia (population 25.6 million) has followed a pathway from a private provider and payer model to a public contract model. ART services were first offered in the 1970s, and while some costs were recoverable from the government for ART-adjacent services (such as hormone assays, ultrasound scans, and clinical procedures), governments only began providing for ART services in the 1990s, with periods of contraction and expansion of funding over the years. As of June 2023, 91 ART clinics are licensed in Australia by the Reproductive Technology Accreditation Committee (RTAC) to provide fertility treatment.^10^ Eighty-seven of these are privately owned and not integrated with a public hospital or institution.^11^ All of these clinics receive some level of public funding through treatment of patients eligible for federal government subsidy under the Medicare Benefits Scheme (Medicare). Corporate conglomeration has led to market concentration among three dominant commercial groups, with an estimated combined market share of over 80 per cent.^12^

There is limited publicly available data in Australia on the number of publicly-funded versus self-funded cycles or the number of cycles provided in the ‘private’ system versus the ‘public’ system. Since the removal of a six-cycle limit in 2000 (and despite recommendations to reinstate the limit),^13^Australia provides unrestricted government subsidy for medically infertile patients undergoing non surrogacy-related fertility treatment.^14^ Patients receiving subsidized cycles still usually pay substantial out-of-pocket costs for services that are not included in the subsidy (such as day hospital fees), and private providers are permitted to charge additional fees above the government rebate. Recently, additional state government programmes have expanded public funding to address some of the out-of-pocket costs associated with IVF, but these are so far limited to specific states and without ongoing commitments to funding in future years.^15^

The funding structure of ART is unusual in both the UK and Australia, though for different reasons. In the UK, especially in England and Wales, the level of self-funded treatment compared to NHS coverage is unlike most other areas of medicine in these countries. In Australia, fertility care is supported by public funding but is commonly delivered by commercial providers, who vastly outnumber ‘public’ clinics integrated into public institutions. This predominance of corporate and commercial clinics that also access public funding (ie, that can access government subsidies as easily as can ‘public’ clinics integrated into public institutions) is also unusual. These complex and varied arrangements operate amongst ongoing debates about the appropriateness of providing public funding for ART.^16^ While these are important issues, the aim of this article is not to make an intervention in debates about what the level of public funding ought to be, though we return to the matter of the public sector in Section V. Our principal purpose is to observe how changing relationships underpinning the delivery of (corporatized) care impact patient care and choice.

### C. Corporate structures in ART

In both Australia and the UK, corporations play a major role in provision of ART. As a ubiquitous economic unit in contemporary capitalist societies, the corporation is a distinct legal entity. In Australia and the UK, the corporation has legal personality akin to a natural person, with many of the same rights and responsibilities. This legal personhood allows companies to engage in commercial activities, such as entering into contracts, owning assets, acquiring loans, and being subject to national taxation schemes. Moreover, corporations enjoy the benefit of limited liability, shielding shareholders from personal liability for the corporation’s actions and finances. This principle of limited liability makes many businesses possible to operate in areas of risk that would deter sole traders and partnerships. In exchange for these advantages, Australian and UK corporations are subject to general company laws and consumer protection laws.

The most prevalent form of corporation among commercial ART clinics is the for-profit corporation limited by shareholders. The business objectives of for-profit ART corporations are not dissimilar from those of other for-profit corporations.^17^ These include the need for revenue and profit generation by, for example, increasing volume or reducing costs, and value creation through provision of additional services or expansion of service offerings.^18^ Clinics may also seek to differentiate themselves from competitors through product differentiation,^19^ such as by making claims about quality, or technological or medical innovation.

The corporate organization of ART has created particular kinds of relationships between doctors and the companies in which they work. In the transition from being an industry consisting of practitioner-owned and largely standalone clinics, to one of corporate conglomerates, many ART clinicians have entered into commercial relationships with large investor-owned management companies and clinic groups. Medical services companies are not new or unique to ART. In other areas of medicine, such as general practice, medical services companies were commonly set up separately from ‘practices’ (ie, the clinician as a sole trader or practice corporation), and the clinician’s practice would receive administrative and medical support services from the medical services corporation and pay a fee for those services under contract.^20^ In contemporary ART, this service relationship appears to be commonly inverted; in Australia clinicians are now often the suppliers of services to the ART clinic company.^21^

### D. Recent commercial and corporate developments

In the last decade, financial investors, such as private equity firms, have shown a strong interest in the fertility sector in both the UK and Australia. Private equity investors seek to generate a relatively short-term return on investment by buying and selling fertility companies within a 3- to 7-year timeframe.^22^ Their approach to improving return on investment often entails reducing the fertility company’s operational costs—eg, through labour reorganization, digitization, or standardization—and consolidating smaller clinics into larger fertility groups. In the IVF sector, this has resulted in a move away from independent smaller clinics run by medical doctors and toward the creation of larger fertility companies with multiple branches of clinics run by more business-oriented boards of managers.^23^ In Australia, some consolidated fertility companies such as Monash IVF are or have been publicly traded on the stock market. Overseas companies are also investing in Australian fertility clinics and vice versa. Virtus Health, for example, is Australia’s largest company and has significant market share in the Danish, Singapore, English, and Irish ART markets.^24^ The reverse has also occurred, with Korean and Singaporean groups purchasing a controlling stake in the Australian company City Fertility in 2018.^25^

In concert with changing patterns of investment, there has been significant vertical integration of fertility products and services. For the corporation, vertical integration involves bringing in associated services in a production chain under common ownership or under a corporate ‘umbrella’. Australian fertility companies, such as Genea and Virtus, for example, are not only clinics, but have in-house pathology, diagnostics, and biotech (eg, gamete banking) departments, to cover the ‘entire IVF journey’.^26^ These products and services are commercially attractive as additional revenue streams for corporations.^27^ Vertical integration may also create supply chain efficiencies, control costs and enable companies to control every aspect of ART.^28^

Alongside bricks and mortar clinics, new online fertility companies have also emerged in recent years. Often started with the aid of capital investment, these fertility companies emphasize the ease and affordability of accessing advice, consultations and tests digitally.^29^ The aspects of the fertility treatment that require physical presence, such as egg retrieval and implantation, are provided through ‘partner clinics’.^30^ At the same time, private insurers often rely on online platforms to manage their networks of affiliate fertility clinics and mediate patient communication—for example through a digital 24/7 ‘concierge’ service.^31^

These ‘corporate’ structures and activities are only one part of the network of financial relationships that make ART a commercial enterprise, but they are highly significant both because of the scale of companies and their control by those with financial agendas. Shareholders (including private equity groups as majority shareholders and controllers of companies) have legitimate influence under corporate law. This has profoundly influenced the web of relationships that shape the delivery of care, and raises the question: what effect do these structures have on patient choice and on the quality of care delivered?

## III. CORPORATIZATION, CHOICE, AND QUALITY OF CARE

Patient choice is an important aspect of good quality care in ART, yet the relationship between corporatization and choice is not a straightforward one. At face value, patient choice appears to increase in a commercialized and corporatized fertility sector because patients have more options. They can choose clinics, include additional products or services on top of ‘routine’ fertility care, select particular payment structures, or choose more actively in relation to treatment continuation or cessation. However, what is at stake is not simply the increase or decrease of choices, but a more complex shift in the nature and context of choice itself.

Although this is often denied, private clinics might put implicit or explicit pressure on clinicians (eg, through protocols or incentives)^32^ to offer treatment to patients for whom it is unlikely to succeed, to recommend more invasive interventions than are necessary (eg, lucrative IVF cycles rather than intrauterine insemination), and to recommend a greater number of cycles (per patient and per clinic) than they otherwise would. Private clinics may also offer a selection of ‘add-on’ interventions (diagnostic and therapeutic interventions that are ‘added on’ to standard IVF cycles) that do not have a sufficient evidence base to be incorporated into standard protocols.^33^ While there are many reasons why patients might want to use add-ons, and why doctors might want to prescribe them (eg, the unique clinical features of a particular case, the desire to innovate, and the desire to respect patients’ autonomy and to keep hope alive^34^) the use of add-ons may also reflect the potential that these technologies hold for increasing revenue per cycle. Importantly, corporate clinics often advertise their services, including add-ons,^35^ thereby creating an expectation among patients that clinicians will offer these services (an expectation that may be difficult for clinicians to resist).^36^ Even where patients do not request particular interventions, clinician suggestions to commence, continue or modify treatments may be received uncritically by patients in health systems in which patients are unused to commercial incentives playing a role in providing care and treatment recommendations.

The idea of patient^37^—or consumer—autonomy is often invoked to justify clinics’ practices. This is sometimes framed as the patient being ‘ultimately responsible for their own treatment choices, provided that they are well informed by their physicians…’^38^ While there is nothing wrong with a commitment to patient autonomy, this can be consciously or unconsciously utilized to justify actions that are influenced by commercial imperatives. It is also important to consider ways in which commercial imperatives can take advantage of the intense desire that some people have to have biologically-related children, and the strongly pro-natalist societies in which patients live. This may create the perception that IVF is a responsibility and/or a need, thereby putting pressure on patients to use IVF in the first place, accept additional interventions and continue trying for success.^39^ Even if the patient is in a good position to decide among possibilities, there may be drawbacks of increasing choice even among ‘good’ options.^40^ Further, good choice requires information to be gathered or provided and understood, which increases the time, effort and ‘psychic costs’ involved (such as asking oneself whether the ‘best’ choice was made).^41^

Even if it was the case that corporatization and commercialization did increase choice, and patients were able to freely decide what they want, choices are not equally accessible to all patients. Most obviously, patients who live in rural or remote areas have limited physical access to services. While this is an issue in both public and private health systems, corporations may be more likely than public providers to ignore or pull services out of locations that are not commercially attractive.^42^

An additional factor impacting on patient access to treatment (and therefore choice) is the consolidation of clinics from independent smaller organizations into larger clinic groups. Streamlining and centralizing services may be beneficial for patients, for example, by increasing convenience and in providing access to several associated services in the one place or at nearby venues. While corporate groups may maintain the separate business identities of acquired clinics (or day hospitals, etc) or internally diversify (such as through the introduction of ‘low cost’^43^ branded clinics within corporate chains or groups),^44^ by definition, conglomeration reduces the number of separately owned competitors by consolidating disparate clinics under common group ownership. This can result both in less choice for patients and reduced competition in the ART marketplace, a key basis upon which limited regulatory intervention upon corporate structures may be justified.^45^ Vertical integration may similarly have negative impacts on patient choice by limiting patients’ ability to choose among providers.

Access to services and patient choice can also be impacted by contractual restraints of trade and non-competition clauses imposed on doctors who contract their services to clinics. Such restraints are common in contracts for services for independent contractors and employment contracts.^46^ On the one hand, mechanisms to restrain trade and enforce non-competition clauses for periods of time aim to protect the legitimate business interests of the clinic if a fertility specialist decides to practice at a rival clinic. On the other hand, restraints of trade can disrupt patient access to their doctor if doctors cannot practice in the area available to existing patients.^47^ This can have even greater consequences where a patient has their gametes or embryos stored with a clinic and their doctor is no longer at that clinic. While transfer of embryos between clinics is not precluded in Australia or the UK, patients can be discouraged in opting for their preferred choice if the process requires advocacy and complex negotiation. Most jurisdictions have strict rules around the enforcement of restraints on trade and in most of Australia, enforceability turns on a test of reasonableness in the circumstances of the case.^48^ In practice, the expense of litigation to challenge restraint of trade clauses likely means that such restraints often go uncontested, or individuals remain in place unaware that such clauses may not be enforceable. Nonetheless, enforcement of restraint of trade raises concerns about patient access to their gametes and embryos, as well as their doctor.^49^

Importantly, even if patients have access to services, this does not mean they can afford them. In this regard, it is noteworthy that there is evidence of inequity in access to publicly subsidized ART. In the UK, access to publicly-funded cycles varies not only between the devolved nations but also between different areas within the devolved nations, in what has been described as a ‘postcode lottery’.^50^ In Australia, the requirements for proving medical infertility^51^ in order to receive Medicare funding have restricted access to subsidized treatment for couples in same-sex relationships and single individuals. While this restriction has been officially lifted, some clinics still rely on the prior definition of medical infertility^52^ creating uncertainty regarding the affordability of IVF services for some people. Additionally, not all patients in Australia have the option of using ‘low cost’ clinics, which often exclude patients above a certain age or body mass index^53^ or ‘complex’ cases, such as those requiring donor gametes. This leaves many people in a situation where they have to pay all costs themselves (in the UK) or considerable out-of-pocket ‘gap’ fees (in Australia), and there are questions about whether the fees charged by private ART providers truly reflect their costs and value, or whether they are simply set by what the market will bear. In this regard, it is noteworthy that in Australia it has been observed that clinics have raised their prices when Medicare rebates are increased.^54^

Rather than lowering their prices, commercial providers may also adopt other strategies to make it easier for patients to ‘afford’ the services. In Australia, some people respond to financial pressures through early access to their superannuation (retirement pensions) to pay for treatment cycles.^55^ In both Australia and the UK, as well as taking out conventional loans to cover the cost of treatment, it is increasingly common for clinics to offer their own consumer credit options, so that patients can undergo private treatment despite being unable to pay for it up-front.^56^ This often-substantial financial risk taken on by patients in the pursuit of parenthood impacts upon other elements of choice because those who are paying for care out-of-pocket may be more receptive to being sold further intervention, such as add-ons, in the (possibly misguided) belief that these will increase the likelihood that they will not need to invest more funds for further cycles.

There are also a range of other, less direct, ways in which corporatization and commercialization can impact negatively on patient care and therefore, on the meaningfulness of choices that patients make. These include loss of continuity of care resulting from mergers and acquisitions or movement of staff^57^ and lack of (perceived) attention and personalization resulting from the use of ancillary staff and standardized protocols^58^ and cutting corners in research and innovation in order to achieve a competitive advantage.^59^

In addition to impacting on the range and quality of patients’ choices, as is the case with any form of organized and regulated health care, corporatized and commercialized provision of ART inevitably shapes the agency and autonomy of clinicians—for example, in determining what ‘add-ons’ a doctor may offer. While working in corporatized ART organizations may offer advantages for doctors, such as financial security and improved work–life balance^60^ as well as access to technological advances and leadership opportunities,^61^ these environments shape the ways in which doctors practice, direct their training and education, influence their knowledge and experience,^62^ and can restrict their freedom to move from one practice to another or to tailor care to individual patient needs or circumstances.

## IV. CURRENT REGULATION OF CORPORATIZED CARE

If we are to minimize the negative impacts of commercialization and corporatization (and harness their strengths), regulation needs to be directed to these issues. At present, ART legislation in both the UK and Australia reflects concerns that dominated at the time of their drafting (in the 1990s and early 2000s) such as human cloning and embryonic experimentation.^63^ To the extent that legislation addresses questions of commercialization, this tends to focus primarily on the commodification of gametes and reproductive processes (eg, in the multi-jurisdictional setting of Australia, where both state and federal-level laws regulate ART, the relevant Act in the state of New South Wales explicitly sets outs an aim to ‘prevent the commercialisation of human reproduction’ but the Act only refers to prohibiting commercial surrogacy^64^). In both the UK and Australia, there are general prohibitions on the sale of gametes.^65^ In contrast, little is said about other aspects of commercialization and corporatization.^66^ In the absence of comprehensive legislation directly addressing commercialization and corporatization, these aspects of the sector are governed primarily through laws and regulations governing the doctor–patient dyad and, to a lesser extent, the clinic–consumer dyad.

### A. The doctor–patient regulatory dyad

The doctor–patient dyad is a key focus of law and regulation in healthcare. The doctor–patient relationship is governed by laws regarding, for example, informed consent, confidentiality, and conflict of interest. In addition to these specific obligations are more general laws and regulations governing the practice of medicine. For example, doctors must achieve standards of care and exercise the proper degree of skill. Both these specific and general regulations may be enshrined or elaborated in professional codes of ethics that are given legal or quasi-legal effect.^67^ However, so far professional codes of ethics have not consistently or substantially addressed corporate and commercial influence on care beyond disclosure and management of financial conflict of interest.^68^ For example, the Australian National Health and Medical Research Council’s *Ethical guidelines on the use of assisted reproductive technology in clinical practice and research* (NHMRC ART guidelines) makes only passing reference to issue of conflict of interest,^69^ and indirectly deals with clinics’ responsibilities via accreditation standards that include both ‘owners’ and patients as stakeholders to whom clinics owe certain information obligations, and whose needs co-define quality.^70^

Despite their apparent influence on care and provision, corporations and their officers are not subject to the same legal and ethical obligations as health practitioners, which are oriented around acting in patients’ best interests. Regulation therefore appears to rely on the assumption that doctors can adequately mediate between patients and the corporate interests of clinic organizations and/or can insulate the doctor–patient relationship, and thereby patient care, from the influence of those interests in provider organizations.

Licensing regimes for provider organizations owned or partially owned by non-practitioners in Australia and the UK are based on a model of a clinic in which a fertility doctor is in charge. In the UK, the ‘Person Responsible’ plays a key role in terms of being accountable to the HFEA,^71^ while in Australia, a ‘Medical Director’ with appropriate specialist qualifications is required for each clinic.^72^ In larger commercial groups, a single individual may be the named person responsible for the licenses for several clinics. This approach, however, simply affirms the centrality of the doctor–patient dyad to ART regulation and assumes that these doctors-in-charge will be able to adequately mediate between patients and the corporate interests of clinic organizations. However, these presumptions may not hold up across the variety of clinic organizational forms (see Table 2), particularly as the scale of the corporate organization increases. Indeed, the HFEA’s recent review seems to confirm that the ‘single clinician as person responsible’ under the clinic licensing regime is insufficiently flexible for modern clinic organizations and protecting patient best interests.^73^

Underpinning both the ‘doctor as overseer’ model of accountability and the professional obligations of doctors to patients more broadly, is a reliance upon the power of health professionals to sufficiently act as ‘filter’ to commercial influences on patient care. There are, however, several dynamics that may prevent this from happening. First, doctors’ engagement with the clinic–corporation allows the corporation to impose code of conduct requirements that reflect its corporate values. For example, Monash IVF Group’s Code of Conduct, as part of the company’s ‘business ethics’, requires doctors to act ‘in the best interests of the Company’, including in their interactions with patients as ‘customers’.^74^ While it is not clear whether such codes have contractual force for doctors engaged by clinics and what the consequences might be, there is no explicit provision for doctors to ensure their clinical independence. Secondly, in changing the ways clinics are organized, the scale of the commercial functions of the organization, and introducing powerful external financial interests, corporatization, and conglomeration can shift the dynamic between the commercial and clinical aspects of providing care. In Australia, clinics rely heavily on doctors to ‘bring’ cycles to the clinic and need doctors for accessing government subsidies.^75^ However, as Adamson and Rutherford (2018) write, ‘when IVF clinics employ multiple physicians and/or provide services in multiple locations, the pressure to be cost-effective and to maximize profit starts to increase. In these large group practice models, revenue generation becomes less dependent on the individual physician or patient’.^76^ This ‘distancing’ increases as the practice group network increases. Thus, there is a scaling effect, where corporate control increases—and doctors lose influence—as the corporate group gets larger. Further, the corporation may be more influential in ART than other areas of medicine, with laboratory and diagnostics services being under corporate control. This suggests that it is naïve to assume that individual practitioners—either as clinician or clinician-manager—can any longer be relied upon as the linchpin of regulation.

### B. The relevance of Consumer law

In addition to professional laws and regulations, generic regulatory frameworks that regulate commerce may afford some protections to patients as ‘consumers’ (with doctors and clinics being providers or vendors of goods or services). Like professional regulation, consumer law is based on a dyad of provider (or ‘vendor’) and consumer, but unlike medical law, consumer law focuses on *directly* governing both corporations and medical professionals as ‘providers’ including not-for-profits.^77^ In the UK, the Competition and Markets Authority (CMA) guidance focuses on clinics and is particularly concerned with the impact of the commercial practices of providers, including non-medical corporate operators, on patient decision making. In Australia, the consumer regulator has highlighted the application of the consumer law to any doctors engaged in private medical practice.^78^ There has been little specific guidance on the application of consumer law to ART beyond voluntary compliance action regarding misleading claims about success rates in advertising,^79^ although consumer guarantees have recently been invoked in an ongoing class action against a large Australian IVF provider group regarding alleged inaccurate testing of embryos.^80^

The clinic–consumer dyad is important in the context of ART because of the ‘distancing’ mechanisms described earlier, and the growth of vertical integration and virtual clinics, as these introduce third parties that interact with patient-consumers but appear disconnected from any medical professionals or licensable (and therefore regulated) ‘brick and mortar’ clinics (see Table 1). In recognition of the important role that consumer law can play, in the UK the CMA has published two sets of guidance, one for clinics on their consumer law obligations,^81^ and one for patients on their consumer rights.^82^ While these documents provide some guidance, it is questionable how much utility they have. Most patients in the UK rely exclusively on the NHS for their medical care, and hence are unused to navigating paid-for treatment services in a context where there may be commercial incentives to oversell and overtreat. Even patients who are more familiar with private medical care may still be unaware of available consumer protections and remedies. Furthermore, even if patients are aware of their rights as consumers, consumer law frameworks do not clearly prescribe prioritization of patient interests and care over other interests.

## V. GOVERNING ART CORPORATIONS

Both professional and consumer regulation are important, but both also fail to address the higher-level tensions that arise between the corporation’s duty to its shareholders, the medical profession’s duty vis a vis the corporations within which they practice, and the obligations of both clinicians and corporations to patients. Addressing the impact of corporate structures and practices on patient care requires us to look beyond the doctor–patient and the provider–consumer dyads, and consider the obligations that corporations and their controllers, and clinicians working in corporate environments, have to patients and health systems. This is important because the ways that corporations make decisions and structure their operations can be legitimate from the business perspective, but they are not necessarily good for patient care.

To better understand this organizational perspective, we can consider models and activities of corporate governance. Corporate governance is the system of rules, practices, policies, and processes by which a company is directed and controlled. It encompasses all aspects of management, including the legitimate bases upon which a corporation may make decisions and structure its operations, and often refers to key principles, including managerial accountability, transparency, responsibility, citizenship, and risk management. Beyond incentives or compliance with laws and regulation broadly, some of these corporate governance principles are legally enshrined for publicly listed companies.^83^ However, these tend to focus on prudential and financial disclosures to address the imbalance of knowledge that disadvantages potential or current shareholders, and to provide assurance to the public market of investment.

In general, there are four key areas of corporate governance with particular relevance to ART. In theory, these are ways in which corporations have attempted to (or been encouraged to) meet a broader set of obligations than simply those owed to shareholders, or to enable those working within them to address tensions between corporate and other (eg, professional) responsibilities. These include through: (i) Stakeholder models of governance and corporate social responsibility; (ii) Corporate-clinical governance mechanisms; (iii) The structure of boards; and (iv) Performance incentives. Each of these, however, has limitations in the healthcare setting.

### A. Stakeholder models and corporate social responsibility

As noted earlier, the most prevalent form of corporation among commercial ART clinics in Australia and the UK is the for-profit corporation limited by shareholders. For-profit corporations are primarily driven by the pursuit of profit maximization, acting in the interests of their shareholders. This is a default position at law in Australia,^84^ where corporate law has traditionally focused on shareholder primacy, meaning directors owe their primary legal duty to the company’s shareholders.^85^ While shareholder primacy still holds some weight in UK law,^86^ a stakeholder model is permitted by company law in the UK. The *Companies Act* 2006 clarified that directors owe duties to the company as a whole, which includes considering the interests of its stakeholders, such as customers and employees.^87^ This appeared to mark a shift towards stakeholder governance in the UK. In 2018, the UK government introduced the *Companies (Miscellaneous Reporting) Regulations 2018*, which require large companies to report on how their directors have considered the interests of stakeholders. Stakeholder governance is also permitted by company law in Australia.^88^ Adoption of a stakeholder model was considered in Australia in a Senate inquiry in 2006, but ultimately reform was not recommended on the understanding that the existing regime allowed for corporations to voluntarily adopt stakeholder governance.^89^

Importantly, stakeholder focussed models make corporate managers able to legitimately consider the interests of stakeholders (such as patients) alongside profits in the discharge of their duties to the company.^90^ The UK has adopted some measures of stakeholder governance in its regulation of corporations by formalizing the principle that good governance involves considering patients as ‘customers’ and framing consideration of their interests squarely within their ‘business relationship’ with the clinic.^91^ However, it is unclear whether clinicians (and governments through the provision of public subsidy for healthcare services), should also be considered ‘stakeholders’, and it is also unclear how tensions between interests of stakeholders (including clinicians, patients, governments and shareholders) should be resolved.^92^ More broadly, there has been little discussion of the uptake of stakeholder models in ART or other healthcare contexts, and some scholars have suggested that stakeholder governance may be difficult to implement in healthcare.^93^ Finally, even if there was a commitment to implementing this model in healthcare, ‘stakeholder benefit’ may be difficult to calculate, and it may not be desirable to have such benefit determined by corporations, especially where market power is concentrated, such as in the Australian ART sector.

More recently, academic attention has focused on another model that attempts to move beyond a sole focus on shareholder profit: corporate social responsibility (CSR). There has been some commentary on CSR in health service organizations, particularly hospitals.^94^ There is substantial scepticism, however, about CSR more broadly,^95^ and whether it results in substantial practical behavioural shifts. Even among healthcare institutions, there is a suggestion that CSR may be framed as an ethics issue in some jurisdictions and as a business marketing issue in others.^96^

Mechanisms such as stakeholder governance and corporate social responsibility may be of limited benefit for patients and health systems especially when they rely on self/co-regulation by businesses, which means that there is little or no external accountability. They also do not create a duty for corporations equivalent to those imposed on health professionals to act in the best interests of patients and do not directly responsibilize corporations for good patient care. More generally, in the health context, they do not address the full range of issues that are addressed by health regulation and therefore cannot simply replace other forms of regulation.

### B. Clinical governance

In many healthcare organizations, devolved organizational clinical governance structures are a corporate governance strategy to manage legal and financial risk and meet obligations to stakeholders. In larger healthcare organizations, clinical governance structures have been viewed as a positive influence of corporatization and conglomeration, providing guidance to doctors in promoting their legal, organizational, and clinical obligations, both to patients and to organizations.^97^ Clinical governance structures have also been portrayed as evidence of an organization’s ‘patient-centred’ approach and clinical rigour.^98^

In Australia, larger ART corporate groups have tended to have internal clinical governance processes, although the form is not consistent.^99^ Despite being seen as a strength of large-scale corporate ART, a recent review of ART in Victoria (the Gorton review) pointed out that these may not be equivalent to clinical governance standards employed in other areas of the health system, and they have been found to be limited in their capacity to promote ongoing safety and quality.^100^ For example, the use of cell-free non-invasive pre-implantation testing (nIPT-A testing)^101^ by the Monash IVF Group has been alleged by patient claimants to have been inaccurate and led to the destruction of embryos.^102^ As a test developed in-house, this case raises more complex concerns around internal governance mechanisms and the management of demands for quality and safety.

Furthermore, within a corporation, clinical governance decisions may be subject to review by the board of directors, the majority of whom are not clinicians. When majority owned by private equity, representatives of that firm are likely to have a board presence. As Braithwaite and Travaglia note, because clinical governance sits within the corporate structure, successful clinical governance requires ensuring that the clinical and corporate governance are linked.^103^ Promoting patient-centred care through clinical governance is dependent on this linkage and it requires active participation, sponsorship and promotion by boards.^104^

### C. Doctors as directors, and directors as doctors

Another common element of corporate governance and structure in healthcare corporations is the appointment of doctors as directors on boards and as senior managers. The engagement of doctors as ‘insiders’ with their operational knowledge and medical training can be seen as an advantage for businesses^105^ and may add legitimacy to a business operating in the healthcare space. Evidence suggests that there may be benefits in terms of organizational financial performance and ‘special competitive insights’,^106^ as well as in terms of clinical quality. For these reasons, the American Medical Association has encouraged doctor participation on boards on the basis of evidence of ‘higher business performance, clinical quality and social outcomes’.^107^

Others, however, have suggested that empirical evidence for the benefits of doctors on boards governing hospitals is mixed.^108^ Furthermore, the presence of a practicing doctor on a board may create conflicts of interest. Unless the company provides a specific carve out for the doctor–director in their constitution, a doctor’s duty to the profession and to their patients may conflict with the doctor’s fiduciary duties as a director. As Borow and others write, ‘considering the inherent tensions and potential conflicts between adhering to the logic of a profit-making, competitive market on one hand and maintaining the doctor’s oath on the other, it is inevitable that dilemmas and difficulties will emerge’.^109^ They also note the parallel between fiduciary duties of directors to companies and the duty of doctors to society (under a ‘social contract’),^110^ while recognizing that doctors also have additional obligations, including a duty to uphold the standards of the profession and to prioritize the best interests of the patients.^111^ In this regard, it is noteworthy that discussions about the benefits of doctors on boards often focus on business benefits, with little engagement with patient care or recognition of the fact that patient interests and choices might conflict with business interests and choices, and whether and how a doctor–director ought to advocate for such at the board level (if at all). Further, there is a question as to whether professional regulation would be effective at the level of a doctor's involvement in management (whether as a director, senior manager, or other agent) where many decisions will be made behind closed doors. It should not be assumed, therefore, that simply appointing a doctor to the board of directors safeguards patients’ interests.

### D. Performance incentives

One corporate governance mechanism of particular relevance to ART is the use of financial incentives, such as contingent remuneration, bonus schemes, and rewarding of company shares, which are intended to motivate performance and align individual interests with company interests. In theory, incentive mechanisms can be used to encourage goods other than maximizing shareholder profits (indeed they can be framed in terms of corporate social responsibility)^112^; however, in many corporations, including in ART, they appear to be used at times to align behaviour with maximizing profit and company financial performance. It is also recognized that such incentives can ‘inadvertently create incentives for unethical behaviour’,^113^ which can be problematic in healthcare contexts.

There are no special rules for corporate managers in corporations operating in the health context, which means that, in ART, directors and senior managers can have at least some of their salary contingent on the company’s financial performance.^114^ In Australia, some ART clinics have offered cycle-based incentives to fertility specialist clinicians.^115^ At the time it was publicly-listed, Virtus, one of Australia’s largest conglomerates, referred to volume-based incentives for IVF cycles for fertility specialists as ‘as a key aspect of Virtus’ business model’.^116^

Despite a broader business management literature linking alignment of interests through incentives to improved financial performance,^117^ there is a lack of empirical work on the actual influence of these cycle-based incentives on clinical and ethical decision making. It seems, however, that cycle-based incentives can create a clear conflict of interest for medical professionals, with a very real risk of leading to the perceived or actual overuse of certain treatments over other less invasive and/or less expensive treatments or non-treatment.^118^

## VI. BOLSTERING THE GOVERNANCE OF MEDICAL CORPORATIONS

The fundamental changes in relationships and care, and clear conflicts in obligations and interests that have been introduced by commercialization and corporatization of ART mean that governance of corporations in this sector must be reconsidered more systematically. Specifically, there is a need to develop regulatory approaches that more explicitly characterize the obligations that corporations (and clinicians working in corporate and commercial environments) have to patients, and that promote fulfilment of these obligations in the face of competing interests and agendas. This entails shifting the focus beyond the doctor-patient dyad in regulation, to reckon with the clinic–patient and clinic–doctor dyads, and to overcome regulatory siloes that diffuse responsibility. Here, we briefly suggest some mechanisms that could improve patient care, quality, and choice, across several domains of governance.

Mechanisms to bolster corporate accountability in the medical context might include:

1. *Expanding licensing schemes* for ART clinics to include a broader range of ‘non-clinical’ considerations, such as, incentive schemes, mergers and acquisitions, research and innovation requirements, and medical involvement in corporate decision making, and to include online clinics under such schemes.
2. *Requiring non-medical directors and senior managers to undergo training* certified by the regulator on medical ethics and law, and, in particular, on ways of preserving medical professionals’ clinical independence when engaging with them.
3. *Attaching rules to public subsidy*, which create incentives/disincentives for clinically—and ethically—sound practice. Here, it is important not to limit the resulting protections to those eligible for subsidy at the risk of creating a two-tiered system of corporate accountability, with different standards applied to public versus private cycles.^119^
4. *Reducing reliance on industry-led regulation* where this is currently the dominant model (eg, in Australia) and *properly empowering independent regulators* to take enforcement action in a manner that suits the nature of the regulated entity (eg, by enabling them to impose financial penalties that do not stop clinics from operating).

Mechanisms to bolster medical accountability in the corporate context might include:

1. *A clearer ban on the offering of cycle-based incentives*, enacted through licensing conditions on clinics or a statutory ban on any person or entity offering volume or cycle-based incentives to medical professionals. This would both do away with the practical requirement of proving unprofessional conduct in Australia, as well as address the negative public perception that financial incentives can create, and clearly reframe such incentives as contrary to law in the medical business.^120^ It is important here that penalties should outweigh the benefit to corporations of noncompliance (ie, of the revenue from additional cycles brought in) and that there is active policing by a regulator rather than relying on complaints of unprofessional conduct.
2. *Contractual or legislative protections for doctors* to fulfil their professional obligations as their first priority, supervening any contractual obligations to ‘act in the best interests of the company and its shareholders.’^121^ This would both protect individual doctors and enable doctor-directors or managers to execute their management and advisory duties by reference to their professional responsibilities (such as to patient care), rather than in the interests of shareholders.
3. *Providing guidance for the medical profession on working in the corporate context.* Guidance may draw upon other areas, for example corporate general practice,^122^ and could provide explicit strategies for dealing with circumstances in which contractual obligations appear to conflict with clinical judgment. There could also be training *provided for doctors to act as directors and senior managers in medical services companies*, as is currently being offered in the USA by the American Medical Association.^123^

Finally, there is a need for a more substantial debate about the place of the ‘public’ sector in assisted reproduction services and the extent to which the dynamic between a robust public offering and private/commercial services markets might have a useful regulatory effect. In this regard, the UK offers a testing ground with a public foundation in the NHS but uneven privatization and increasing outsourcing in recent years. Australia, in contrast, illustrates the manner in which ART develops where corporatization is advanced, and in a system without a substantial public-provider sector of ART but with substantial public-subsidy. Further, it may be possible to address some of the challenges of corporatization by considering the potential regulatory effects of other types of private but non-profit organizations.^124^

There might be constitutional and other barriers to these proposed mechanisms, and some will be more relevant in some jurisdictions than others—for example, the use of public subsidization as a mechanism may be more suitable to Australia, where corporate clinics receive substantial subsidy, whereas in the UK, rules for publicly funded cycles would only apply to the minority of cycles in practice, as most are patient self-funded. Similarly, changes in the balance of external versus industry self-regulation may be particularly salient in Australia, where there is a concentration of clinics among three large commercial groups. A national independent regulator in the ART space (or for medical services companies more broadly) might also overcome the patchwork of state-based regimes that have developed in ART amid only a partially federated approach to health regulation in Australia.^125^

Nonetheless, there is evidence that both Australia and the UK have an appetite for reform. In the UK, the HFEA has proposed changes to legislation that would permit imposing financial penalties on businesses that are non-compliant with licensing conditions and recommended that the definition of licensable clinics be expanded to capture online ‘clinics.’^126^ In Australia, the Gorton review, one of a number of state-based reviews, made several recommendations in acknowledgement of ‘an increasingly corporate and competitive approach to service provision’ more broadly.^127^ There have also been efforts (albeit unsuccessful) to limit the number of publicly funded cycles.^128^

## VII. CONCLUSION

Corporatization and commercialization in ART clearly impact patient choice, but it is questionable whether this impact is entirely positive. It is crucial that regulation of ART supports meaningful and autonomous choice. However, regulation in ART reflects presumptions about a doctor–patient-centric model of provision that is increasingly inconsistent with the way that ART is actually provided. Now we are reckoning with the impact of large-scale globalized, commercialized, and corporatized service markets on fertility patients and medical practice. Fundamental changes in relationships and care, the expanding scope of activities by non-medical actors, and clear conflicts in obligations and interests mean that governance must more explicitly characterize the obligations that corporations, and clinicians working in corporate and commercial environments, have to patients, and to promote fulfilment of these obligations in the face of competing interests and agendas. To put this another way, the notion that ‘what’s good for patients is good for business’ is an oversimplification and there is need to be sceptical about such claims as a defence against regulatory oversight and/or to protect status quo self-regulation. Patient interests and business interests are not automatically aligned. Organizations will largely not have malicious intent, but there is risk of patient care being deprioritized amid tensions between the obligations of decision makers.

Furthermore, the corporate forms and financial developments that we have described suggest that neat distinctions between the clinical and the non-clinical (administrative or ‘business’) elements in ART are not sustainable in practice. Indeed, the entanglement of the clinical and the commercial is rooted in the very structure of the corporate service, from the level of organizational management through to interactions with patients. Further, the clinical and the commercial are used to frame one another, such as through positioning commercial providers as responders to patient demand and enablers of autonomy via choice, while representing business and patient interests as aligned (for example, as a foil to accountability). It is, therefore, not just the ‘who is being regulated’ that is out of date, but also the ‘what is being regulated’—which is not simply co-existing clinical and business structures, but rather an entangled clinical and commercial structure.

In order, therefore, to protect and improve patient care and reproductive choice in ART, we must move beyond asking whether commercialization is ‘good’ or ‘bad’ for practice and instead address the ways in which the commercial and the clinical intersect. Regulation of corporate medicine thus requires that we recognize both the distinctions between the clinical and the commercial, and the ways in which they rely upon and influence each other. It is the tension between the need for insulation and integration that is at the core of the regulatory problem.

## DECLARATIONS

Isabel Karpin is a member of the journal's Editorial Advisory Board.

Ian Kerridge was an author of the Australian National Health and Medical Research Council’s *Ethical guidelines on the use of assisted reproductive technology 2017*.

Emily Jackson was part of the UK Human Fertilisation and Embryology Authority’s Legislative Reform Advisory Group.

## Footnotes

1 Eg, M Brazier, ‘Regulating the Reproduction Business?’ (1999) 7 Medical Law Review 166; DL Spar, *The Baby Business: How Money, Science, and Politics Drive the Commerce of Conception* (Harvard Business Press 2006).

2 Corporatization is not unique to ART and has been observed and critiqued across several areas of medicine, including general practice, hospitals, dentistry, radiology, and pathology. These have also undergone conglomeration. See eg, K White and F Collyer, ‘To Market, To Market: Corporatisation, Privatisation and Hospital Costs’ (1997) 20 Australian Health Review 13; F Collyer and K White, *Corporate Control of Health Care in Australia* (Discussion Paper 42, The Australia Institute 2001); C de Moel-Mandel and V Sundararajan, ‘The Impact of Practice Size and Ownership on General Practice Care in Australia’ (2021) 214 Medical Journal of Australia 408; A Holden, L Adam and W Thomson, ‘Rationalisation and “McDonaldisation” in Dental Care: Private Dentists’ Experiences Working in Corporate Dentistry’ (2021) British Dental Journal. <https://www.nature.com/articles/s41415-021-3071-3> accessed 6 June 2024.

3 E Docteur and H Oxley, ‘Health-Care Systems: Lessons from the Reform Experience’ OECD Economics Department Working Paper 374 (OECD Publishing 2003) 7.

4 ibid 10.

5 Warnock Committee, *Report of the Committee of Inquiry into Human Fertilisation and Embryology* (HMSO 1984) para 5.11.

6 Human Fertilisation and Embryology Authority, *State of the Fertility Sector 2022/23* (Annual report, 2023).

7 Human Fertilisation and Embryology Authority, *Fertility Treatment 2021: Preliminary Trends and Figures* (2023).

8 ibid.

9 Department of Health and Social Care, *NHS-funded In Vitro Fertilisation (IVF) in England* (Department of Health and Social Care 2023).

10 In Australia, these figures are only reported annually. Reproductive Technology Accreditation Committee, *Annual Report 2022–2023* (2023).

11 Four of these registered IVF clinics are within public hospitals in Australia and offer fertility treatment beyond oncofertility programmes: New South Wales Department of Health, ‘Improving Affordability and Access to IVF and Fertility Services in NSW—Maternal and Newborn’ <https://www.health.nsw.gov.au : 443/affordable-ivf> accessed 21 December 2023; The Royal Women’s Hospital, ‘Public Fertility Care Service’ <https://www.thewomens.org.au/patients-visitors/clinics-and-services/fertility-genetics/public-fertility-services> accessed 21 December 2023.

12 Although it is unclear whether cycles conducted in the public system are included in this reporting. IBISWorld, *Fertility Clinics in Australia* (Industry Report, December 2023) 36.

13 Australian Government Department of Health, Medicare Benefits Schedule Review Taskforce: Taskforce Report on Gynaecology MBS Items (2020) (Australian Government Department of Health, Canberra) 25.

14 ibid 38.

15 Office of the Premier of Victoria, ‘Public IVF to Make Starting a Family Easier for Victorians’ <http://www.premier.vic.gov.au/public-ivf-make-starting-family-easier-victorians> accessed 1 December 2023; New South Wales Department of Health (n 11).

16 See P Mladovsky and C Sorenson, ‘Public Financing of IVF: A Review of Policy Rationales’ (2010) 18 Health Care Analysis 113.

17 GD Adamson and AJ Rutherford, ‘The Commercialization of In-Vitro Fertilization’ in G Kovacs, P Brinsden and A DeCherney (eds), *In-Vitro Fertilization: The Pioneers’ History* (CUP 2018) 240–48.

18 ibid.

19 ibid.

20 de Moel-Mandel and Sundararajan (n 2); Australian Medical Association, *Corporatisation of General Practice Decision Support Kit for Doctors* (AMA 2015).

21 See Virtus Health Limited, *Prospectus* (24 May 2013); see also *Australian Competition and Consumer Commission v IVF Finance Pty Limited (No 2)* [2021] FCA 1295, [28] cf [22]. <https://announcements.asx.com.au/asxpdf/20130611/pdf/42gdgl8tfbz0l5.pdf> accessed 13 December 2023; Monash IVF, *Prospectus* (13 June 2014) https://announcements.asx.com.au/asxpdf/20140626/pdf/42qg079q7nj4jy.pdf> accessed 13 December 2023.

22 JM Zhu and D Polsky, ‘Private Equity and Physician Medical Practices—Navigating a Changing Ecosystem’ (2021) 384 New England Journal of Medicine 981, 981. Private equity investment has been described as part of the ‘financialization’ of the fertility sector through the capital investments in ART and the approximation of the financial and fertility industries: L Van de Wiel, ‘The Speculative Turn in IVF: Egg Freezing and the Financialization of Fertility’ (2020) 39 New Genetics and Society 306, 311.

23 P Patrizio and others, ‘The Changing World of IVF: The Pros and Cons of New Business Models Offering Assisted Reproductive Technologies’ (2022) 39 Journal of Assisted Reproduction and Genetics 305, 308.

24 A Stuhmcke, ‘Reframing the Australian Medico-Legal Model of Infertility’ (2021) 18 Bioethical Inquiry 305, 311.

25 ibid 312; S Thompson, A MacDonald and J Moullakis, ‘City Fertility Sells Majority Stake to Korean-Led Consortium’ (Australian Financial Review, 31 January 2018) <https://www.afr.com/street-talk/city-fertility-sells-majority-stake-to-asiabased-consortium-20180131-h0rkaz> accessed 24 January 2023.

26 L van de Wiel, ‘The Datafication of Reproduction: Time‐lapse Embryo Imaging and the Commercialisation of IVF’ (2019) 41 Sociology of Health and Illness 193, 204.

27 Patrizio and others (n 23) 307.

28 FL Saleh and others ‘Changes to Reproductive Endocrinology and Infertility Practice, Research and Training as Investor Mergers Increase’ F&S Reports (2023).

29 See eg, Apricity Fertility UK, ‘Apricity Fertility Clinic: The UK’s Top Virtual IVF Clinic’ <https://www.apricityfertility.com/uk> accessed 21 December 2023.

30 Apricity Fertility UK, ‘Apricity Fertility Clinic: How It Works—Your Path to Parenthood’ <https://www.apricityfertility.com/uk/why-apricity/how-it-works> accessed 21 December 2023.

31 L van de Wiel, ‘Disrupting the Biological Clock: Fertility Benefits, Egg Freezing and Proactive Fertility Management’ (2021) 14 Reproductive Biomedicine & Society Online 239.

32 We discuss the example of IVF cycle volume-based financial incentives for fertility specialists offered by corporate clinics in Section IV.

33 S Lensen and others, ‘How Common Is Add-on Use and How Do Patients Decide Whether to Use Them? A National Survey of IVF Patients’ (2021) 36 Human Reproduction 1854; ESHRE Add-ons working group and others, ‘Good Practice Recommendations on Add-ons in Reproductive Medicine’ (2023) 38 Human Reproduction 2062.

34 See A Wrigley and others, ‘Hope and Exploitation in Commercial Provision of Assisted Reproductive Technologies’ (2023) 53 Hastings Center Report 30.

35 See L van de Wiel and others, ‘The Prevalence, Promotion and Pricing of Three IVF Add-Ons on Fertility Clinic Websites’ (2020) 41 Reproductive BioMedicine Online 801.

36 See O Iacoponi and others, ‘Passion, Pressure and Pragmatism: How Fertility Clinic Medical Directors View IVF Add-Ons’ (2022) 45 Reproductive BioMedicine Online 169; C Stanbury and others, ‘What Moral Weight Should Patient-Led Demand Have in Clinical Decisions about Assisted Reproductive Technologies?’ (2024) 38 Bioethics 69.

37 We note that the use of the terms ‘patient’ and ‘consumer’ in the context of ART are contested. In this article, we use the term ‘patient’ to refer to any person who seeks to use ART. We use the term ‘consumer’ when specified as such by relevant sources or in law. For example, in discussions of consumer law.

38 O Iacoponi and others (n 36) 172.

39 A Petropanagos, ‘Pronatalism, Geneticism, and ART’ (2017) 10 International Journal of Feminist Approaches to Bioethics 119.

40 G Dworkin, ‘Is More Choice Better than Less?’ in G Dworkin (ed), *The Theory and Practice of Autonomy* (CUP 1988) 62–81.

41 ibid 67.

42 A Sassano and others, ‘Going the Distance’ (2023) 20 Journal of Bioethical Inquiry 225

43 In Australia, there are also ‘bulk-billed’ IVF clinics, where providers do not charge out-of-pocket costs above government rebates.

44 For eg, The Fertility Centre offers ‘affordable IVF’ at ‘a fraction of what you would pay at other IVF clinics’ and is part of the Virtus Health clinic group: The Fertility Centre, ‘IVF Costs & Fees’ (*The Fertility Centre*) <https://www.thefertilitycentre.com.au/ivf-costs> accessed 30 November 2023.

45 An example of this is the Australian Competition and Consumer Commission (ACCC) intervention on the sale of the Adora ‘low cost’ clinics and day hospitals to Virtus Group on the basis that it would substantially lessen competition in contravention of the Australian Consumer Law. See *Australian Competition and Consumer Commission v IVF Finance Pty Limited (No 2)* [2021] FCA 1295.

46 Enforceability of a restraint of trade clause in a contract for services was the subject of dispute between a fertility specialist and a fertility clinic in *Monash IVF Pty Ltd v Burmeister* [2017] NSWSC 849.

47 M Han, ‘Dr Lynn Burmeister Says Monash IVF’s One-Year Restraint “Unenforceable”’ (*Australian Financial Review*, 16 July 2017) <https://www.afr.com/companies/healthcare-and-fitness/dr-lynn-burmeister-says-monash-ivfs-oneyear-restraint-unenforceable-20170712-gx9jbr> accessed 20 June 2023.

48 See *Sidameneo (No 456) Pty Ltd v Alexander* [2011] NSWCA 418, [3]-[126] (Young JA). In New South Wales, restraint of trade clauses are presumed to be valid to the extent they are not against public policy: s 4 *Restraints of Trade Act* 1976 (NSW).

49 J Sinnerton, ‘“Don’t have a Doctor to Talk To”: Claims Embryos Held Hostage as IVF Clinic Gutted of Specialists’ (*Courier Mail*, 26 September 2022) <https://www.couriermail.com.au/news/queensland/embryos-held-hostage-as-brisbane-ivf-facility-gutted-of-specialists/news-story/04fff46c53e93cdf6ba0adedf615ea83> accessed 30 November 2023.

50 British Pregnancy Advisory Service, ‘BPAS Investigation into the IVF Postcode Lottery: An Examination of CCG Policy for the Provision of Fertility Services’ (*BPAS*, 2020) <https://www.bpas.org/media/3369/bpas-fertility-ivf-postcode-lottery-report.pdf> accessed 20 December 2023.

51 A service must be ‘clinically relevant’ in order to receive government subsidies: ss 3 and 20(1) *Health Insurance Act* 1973 (Cth). In ART, clinical relevance is determined by the ‘medical profession as necessary to appropriately treat a patient’s medical infertility’: Services Australia, ‘Education Guide—Billing Assisted Reproductive Technology Services—Services Australia’ <https://www.servicesaustralia.gov.au/education-guide-billing-assisted-reproductive-technology-services> accessed 29 August 2023.

52 N Evans ‘IVF’ (*Lesbians and the Law*) <https://www.lesbiansandthelaw.com/ivf> accessed 18 January 2024.

53 See eg, First Step Fertility, ‘Bulk-Billed IVF: Are We Eligible?’ <https://www.firststepfertility.com.au/bulk-billed-ivf-am-i-eligible/> accessed 22 November 2023.

54 GM Chambers and others, ‘A Reduction in Public Funding for Fertility Treatment—An Econometric Analysis of Access to Treatment and Savings to Government’ (2012) 12 BMC Health Services Research 142.

55 N Bhatia and L Porceddu, ‘Emptying the Nest Egg to Fill the Nursery: Early Release of Superannuation to Fund Assisted Reproductive Technology’ (2021) 44 UNSW Law Journal 513.

56 Although facilitating the debt-financing of IVF treatment might make it accessible to more people, there may be inevitable conflicts of interest if doctors are recommending treatments to patients, at the same time as offering them a credit facility to pay for them. Poor prognosis patients thus become a potentially lucrative source of revenue: MB Jacoby, ‘The Debt Financing of Parenthood’ (2009) 72 Law and Contemporary Problems 147.

57 See eg, Sinnerton (n 49).

58 S Gallagher and others, ‘Medicine in the Marketplace: Clinician and Patient Views on Commercial Influences on Assisted Reproductive Technologies’ (2024) Reproductive BioMedicine Online (forthcoming).

59 In Australia, there is an ongoing class action lawsuit concerning the provision by Monash IVF of cell-free non-invasive pre-implantation genetic testing (niPGT-A testing) of patient’s live embryos. The claim will argue that the testing had a substantial risk of false positive that was not disclosed to the patients. While the matter is still to be determined, the existence of the claim raises the spectre that suggests that commercial imperatives might be even leading clinics to rush processes of research and innovation, and mislead patients about the harms and benefits of interventions, even if Monash IVF are able to show that that is not what happened in this case: ‘Second Amended Statement of Claim’, *Bopping and Pedersen v Monash IVF Pty Ltd and others* (S ECI 2020 04761, Supreme Court of Victoria, 18 August 2023).

60 de Moel-Mandel and Sundararajan (n 2) 408.

61 S Gallagher and others (n 58).

62 Patrizio and others (n 23).

63 In the UK: Human Fertilisation and Embryology Authority, *Modernising Fertility Law—Recommendations from the Human Fertilisation and Embryology Authority (HFEA) for changes to the Human Fertilisation and Embryology Act 1990* (2023). In Australia, the focus of federal legislation that introduced the national fertility clinic licensing regime was to address community concerns, including ethical concerns, about scientific developments in relation to human reproduction and the use of human embryos in research activities: see *Research Involving Human Embryos Act 2002* (Cth) s 3 and *Prohibition of Human Cloning for Reproduction Act 2002* (Cth) 3. See also C Mayes, ‘Co-producing Bioethics: How Biomedical Scientists and Applied Philosophers Established Bioethics in Australia’ (2022) 35 Social History of Medicine 1310.

64
*Assisted Reproductive Technology Act 2007* (NSW). In Australia, some states such as New South Wales, have specific legislation for fertility treatment. The limited legislative powers of the Australian Commonwealth over health mean that state-based legislation for health matters is common. See also Surrogacy Act 2010 (NSW), s 8.

65 Though limited payments are permitted to be made to donors to compensate for expenses incurred in the course of providing a donation: *Human Fertilisation and Embryology Act 1990* (UK) s 12(1)(e); *Prohibition of Human Cloning for Reproduction Act 2002* (Cth) s 21.

66 However, the Gorton review in the state of Victoria acknowledged the increased influence of corporate clinic organizations and ‘changing drivers for demand’: M Gorton, *Helping Victorians Create Families with Assisted Reproductive Treatment—Final Report of the Independent Review of Assisted Reproductive Treatment* (Victorian Government, May 2019) 191.

67 For example, Australian courts refer to and apply respective codes of professional ethics as evidence of what constitutes appropriate professional conduct or practice: s 41 *Health Practitioner Regulation National Law Act 2009* (Cth). See eg, *Panegyres v Medical Board of Australia* [2020] WASCA 58.

68 See B Blakely and others, ‘Conflicts of Interest in Australia’s IVF Industry: An Empirical Analysis and Call for Action’ (2019) 22 Human Fertility 230.

69 Australian National Health and Medical Research Council, *Ethical Guidelines on the Use of Assisted Reproductive Technology in Clinical Practice and Research 2017* (NHMRC 2023) 28.

70 Fertility Society of Australia and New Zealand, *Reproductive Technology Accreditation Committee Code of Practice for Assisted Reproductive Technology Units* (October 2021) ss 1.2 and 4.

71
*Human Fertilisation and Embryology Act 1990* (UK), s 17.

72 Fertility Society of Australia and New Zealand (n 70) s 1.4.1. Clinic accreditation by the Reproductive Technology Accreditation Committee of the Fertility Society of Australia is required under section 8, *Research Involving Human Embryos Act 2002* (Cth).

73 Human Fertilisation and Embryology Authority (n 63).

74 Monash IVF Group Limited, ‘Code of Conduct 2023’ <https://assets.monashivf.com/assets/Code-of-Conduct_Board-approved-6.3.2023.pdf> accessed 13 December 2023.

75 Under the Health Insurance Act 1973 (Cth), which underpins the Medicare Benefits Scheme, Medicare benefits are typically only payable for services rendered by or on behalf of an appropriately registered practitioner: see ss 3(1), 17, 19, 19AA.

76 GD Adamson and AJ Rutherford, ‘The Commercialization of In-Vitro Fertilization’ in G Kovacs, P Brinsden and A DeCherney (eds), *In-Vitro Fertilization: The Pioneers’ History* (CUP 2018) 240–48.

77 In Australia, consumer law applies to any person acting in trade or commerce with consumers, including not for profits: see *Competition and Consumer Act 2010* (Cth) sch 2. In the UK, the Competition and Markets Authority takes the view that consumer law applies to clinics in relation to the provision of any treatment that is paid for by a patient, even if care is partially subsidized by the NHS, and regardless of whether the clinic is an NHS or private clinic, and whether it is run for-profit, or not-for-profit. Consumer law can also apply to other businesses having dealings with consumers in the fertility sector: Competition and Markets Authority, *A Guide for Clinics* (CMA 2021) paras 2.14–2.20.

78 Australian Competition and Consumer Commission, ‘Medical Professionals’ (1 May 2023) <https://www.accc.gov.au/business/competition-and-exemptions/associations-and-professional-services/medical-professionals> accessed 20 July 2023.

79 In 2016, the Australian Competition and Consumer Commission took regulatory action to address misleading industry practices in advertising of services and success rates following a complaint from the Australian Health Practitioner Regulation Agency. See Australian Competition and Consumer Commission, ‘IVF “success Rate” Claims under the Microscope’ (Media Release 212/16, 14 November 2016) <https://www.accc.gov.au/media-release/ivf-success-rate-claims-under-the-microscope> accessed 20 July 2023.

80
*Competition and Consumer Act 2010* (Cth) sch 2 (the ‘Australian Consumer Law’ or ACL) provides for several consumer guarantees (among other consumer protections), including that services will be provided with due care and skill, and be fit for purpose: ss 60, 61. Remedies under the ACL include repair, replacement, refund and compensation: pts 5.2 and 5.4. This class action represents a rare instance of consumer law being tested in the context of ART, and also raises the issues of damages for psychiatric injury. See ‘Second Amended Statement of Claim’, *Bopping and Pedersen v Monash IVF Pty Ltd and others* (S ECI 2020 04761, Supreme Court of Victoria, 18 August 2023). Note that any medical professionals who may have advised and been part of informed consent processes had not been joined to proceedings at the time of writing this article.

81 Competition and Markets Authority (n 77).

82 Competition and Markets Authority, *A Guide to your Consumer Rights* (CMA 2021).

83 For eg, in Australia publicly-listed entities are required to benchmark their corporate governance practices against the principles and recommendations of the ASX Corporate Governance Council: Australian Securities Exchange, *Listing Rules* (1 December 2019) r 4.10.3.

84 It might be that healthcare sector was not specifically contemplated by the Inquiry—this is reflected in the lack of submissions and discussion about corporations in the healthcare or medical services industry: Australian Government Parliamentary Joint Committee on Corporations and Financial Services, *Corporate responsibility: Managing risk and creating value* (Commonwealth of Australia, June 2006).

85 This is enshrined in s 181 of the *Corporations Act 2001* (Cth), which requires directors to act in good faith in the best interests of the company. Although, recent years have seen an increasing recognition of the importance of stakeholders in corporate governance. For example, the Financial Services Royal Commission (2018–2019) highlighted the need for companies to consider the interests of a broader range of stakeholders, including customers, employees, and the community: K Hayne, *Royal Commission into Misconduct in the Banking, Superannuation and Financial Services Industry* (Final Report, 2019).

86
*Companies Act 2006* (UK) s 172.

87 As part of what is referred to in the Explanatory Notes as the principle of ‘enlightened shareholder value’: *Companies Act 2006* (UK) s 172 and Explanatory Notes, para 325.

88 See Australian Government Parliamentary Joint Committee on Corporations and Financial Services (n 84).

89 ibid. The 2006 Inquiry did not address the question of how many companies actually had voluntarily adopted a stakeholder approach, and barriers to adopting such a model.

90 See VH Ho, ‘“Enlightened Shareholder Value”: Corporate Governance Beyond the Shareholder-Stakeholder Divide’ (2010) 36 Journal of Corporation Law 59.

91
*Companies Act 2006* (UK) s172(1).

92 Ho (n 90). See also JM Karpoff, ‘On a Stakeholder Model of Corporate Governance’ (2021) ECGI Working Paper Series in Finance 749/2021, 24–25.

93 D Jamali, M Hallal and H Abdallah, ‘Corporate Governance and Corporate Social Responsibility: Evidence from the Healthcare Sector’ (2010) 10 Corporate Governance: The International Journal of Business in Society 590.

94 For eg, C Brandão and others, ‘Social Responsibility: A New Paradigm of Hospital Governance?’ (2013) 21 Health Care Analysis 390.

95 See RC Kim, ‘Rethinking Corporate Social Responsibility under Contemporary Capitalism: Five Ways to Reinvent CSR’ (2022) 31 Business Ethics, the Environment & Responsibility 346.

96 G Tomaselli and others, ‘Healthcare Systems and Corporate Social Responsibility Communication: A Comparative Analysis Between Malta and India’ (2018) 26 Journal of Global Information Management 52.

97 Gallagher and others (n 58).

98 For eg, Monash IVF Group Limited, *Annual Report 2021* (2021) 21.

99 Gorton (n 66) x.

100 ibid.

101 Not to be confused with noninvasive prenatal testing (NIPT), which refers to methods of assessing placentally derived DNA circulating in the bloodstream of the pregnant individual, to determine the chance a fetus has certain chromosomal or genetic conditions via assessing.

102 ‘Second Amended Statement of Claim’, *Bopping and Pedersen v Monash IVF Pty Ltd and others* (S ECI 2020 04761, Supreme Court of Victoria, 18 August 2023). See also Bopping (n 59).

103 J Braithwaite and JF Travaglia, ‘An Overview of Clinical Governance Policies, Practices and Initiatives’ (2008) 32 Australian Health Review: A Publication of the Australian Hospital Association 10.

104 ibid.

105 See eg, M Borow and others, ‘The Physician on a Board of Directors: Bane or Benefit?’ (2022) 14 Journal of Healthcare Leadership 17.

106 RN Miller, ‘Physicians Encouraged to Take Seat at Table on Health Care Boards’ (*AMA*, 12 June 2017) <https://www.ama-assn.org/practice-management/scope-practice/physicians-encouraged-take-seat-table-health-care-boards> accessed 15 December 2023.

107 ibid. See American Medical Association, *Proceedings of the 2017 Annual Meeting of the American Medical Association House of Delegates* (2017) 356, Policy H-405.990.

108 L Bennington, ‘Review of the Corporate and Healthcare Governance Literature’ (2010) 16 Journal of Management and Organization 314.

109 M Borow and others (n 105) 18.

110 The obligations of doctors to society, and corporations to society, are raised in literature on medical professional ethics and corporate governance and social responsibility, respectively, as are concepts of ‘social contract’. However, these are beyond the scope of this article.

111 For eg, in Australia, under the *Health Practitioner Regulation National Law*.

112 See eg, GB Derchi, L Zoni and A Dossi, ‘Corporate Social Responsibility Performance, Incentives, and Learning Effects’ (2021) 173 Journal of Business Ethics 617.

113 Institute of Business Ethics UK, ‘IBE Guidance for Board Members on Developing an Ethical Business Culture’ (2 October 2023) <https://www.ibe.org.uk/resource/ibeboardguidance.html> accessed 21 December 2024.

114 For example, in their FY21 Annual Report, the then-publicly listed Australian IVF clinic group Virtus Health stated their remuneration strategy as ‘reward[ing] executives for achievement and over-achievement of short and long-term objectives to align executives with shareholders’ interests through a mix of fixed and variable remuneration components.’: Virtus Health, *Annual Report 202*1 (2021) 29.

115 ibid. Further detail on the incentive schemes is available in financial reporting to the Australian Securities Exchange as a requirement of public listing: see Virtus Health Limited, *Appendix 4E Preliminary Final Report* (2020) 83.

116 Virtus Health Limited (n 21) 38.

117 See SJ Perkins and C Hendry, ‘Ordering Top Pay: Interpreting the Signals’ (2005) 42 Journal of Management Studies 1443, 1446; M Vartiainen and others, *Reward Management-Facts and Trends in Europe* (Pabst Science Publishers 2008); AJ Nyberg and others, ‘Agency Theory Revisited: CEO Return and Shareholder Interest Alignment’ (2010) 53 Academy of Management Journal 1029.

118 On top on existing pressures to over-treat using IVF in the commercial environment: GD Adamson and AJ Rutherford, ‘The Commercialization of In-Vitro Fertilization’ in G Kovacs, P Brinsden and A DeCherney (eds), *In-Vitro Fertilization: The Pioneers’ History* (CUP 2018) 242.

119 A further consideration in Australia is the application of any rules upon users of the Medicare Benefits system. At present, claiming benefits is limited to registered practitioners. In practice, clinics can process these claims as ‘administrators’ on behalf of practitioners (who in turn, are claiming the benefit on behalf of patients). To be effective in enacting corporate responsibility, the public funding lever would need to directly responsible entities that interact with the claims system. Alternatively, legislative change to apply rules to any entity that provides a subsidized service may be appropriate.

120 In Australia, incentives have continued to be offered despite existing penalties for inciting ‘unprofessional conduct’. While volume-based financial incentives can be legitimate from the perspective of financial managers, they undermine professional ethics, and may be contrary to law. For example, in Australia, the *Health Practitioner National Law* (HPNL) regulates health professionals against accepting benefits for referral or recommendations as unprofessional conduct: see *Health Practitioner Regulation National Law Act 2009* (NSW) s 136B. The HPNL also appears to regulate ART clinics incidentally against the use of incentives if these amount to directing or inciting unprofessional conduct, resulting in fines of up to AUD$60,000 for an individual or $120,000 for a corporation: *Health Practitioner Regulation National Law Act 2009* (NSW), s 136 (see also mirror provisions enacted in each state and territory as part of the ‘adoption of laws’ model to legislating the HPNL).

121 See eg, Monash IVF Group (n 74). Though we note it is unclear whether the Monash IVF code of conduct would be incorporated into contracts for services.

122 The Australian Medical Association provided a corporatisation ‘decision support kit’ for general practitioners in 2015, amidst a trend of corporatisation and conglomeration in general practices. In addition to considerations when selling a practice to a corporate group, the toolkit points practitioners to considerations when working ‘under the corporate umbrella’ and suggests, for example, considering whether ‘any contractual obligations have the *potential* to impact adversely on …[clinical] independence’ (emphasis added): Australia Medical Association, *Corporatisation of General Practice—Decision Support Kit for Doctors* (AMA 2015) 29.

123 See Borow and others (n 105).

124 As well as their viability in the sector. For eg, the now closed BPASS UK clinic was run by a charity and overseen by a board of trustees.

125 In Australia, any national-level regulation of fertility treatment or assisted reproductive technologies may require states to devolve their power to the federal government to avoid an absence of Commonwealth Constitutional authority.

126 Human Fertilisation and Embryology Authority (n 63).

127 Gorton (n 66) 7.

128 See Australian Government Department of Health (n 13).

